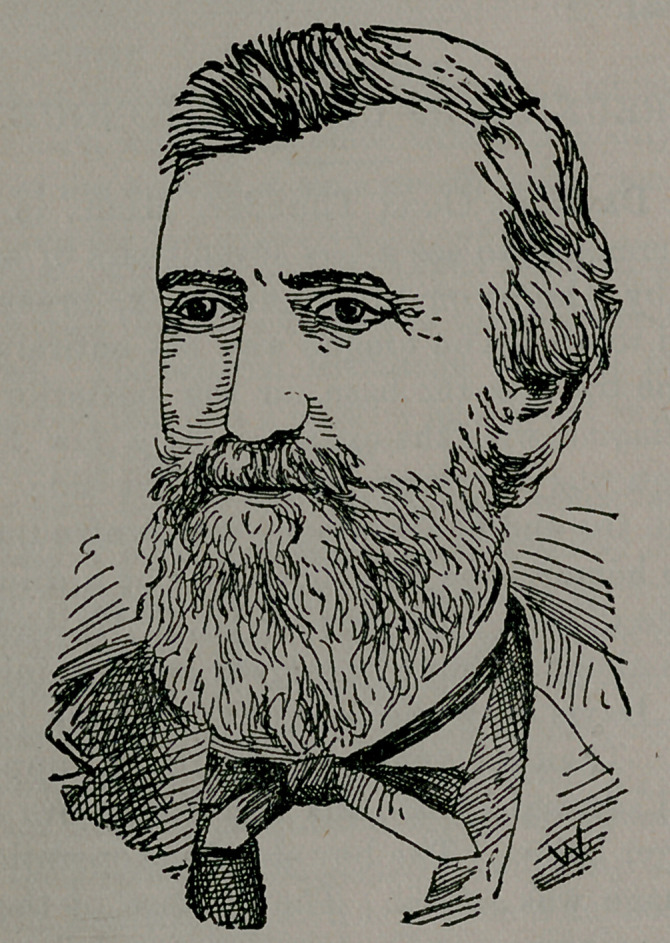# In Memory of Dr. Robert Campbell Word

**Published:** 1890-10

**Authors:** Thos. S. Powell


### IN MEMORY OF DR. ROBERT CAMPBELL WORD.

Among the labors of love given to us as a grateful privilege may be classed that of paying tribute to the memory of a good and great man, whom we have known intimately and loved as a dear and valued friend. Such was the subject of this sketch to the writer, who, with both sadness and pleasure, commemorates the many virtues of his late associate in the faculty of the Southern Medical College.

Dr. Word was born in Laurens District, South Carolina, January 27th, 1825, and died July 26th, 1890.

When only three years of age he lost his father. His mother, left with a large family of children, settled in Decatur, Georgia, and remained one year, then settled in Cassville, Georgia, where Dr. Word was reared and educated. His medical preceptor was the well known Dr. A. L. Barry.

He was a student of the Georgia Medical College at Augusta, Ga., in 1845–46, and in the spring of 1847 graduated with honors at the Medical University of New York. He then returned to Cassville and began the practice of medicine. Shortly afterwards he was married to Miss Adeline Patton, with whom his wedded life was a long and happy one to the date of his death

A few years after his marriage he removed to Rome, Ga., where he rose to a prominence and popularity that made him a leader in the profession. There he remained until 1862, when he entered the Confederate army as a surgeon, and served in that capacity to the close of the war.

In 1866 Dr. Word moved to Atlanta, where he soon became prominent before the public and in the profession, and won many friends by his genial and loveable nature and sterling, manly virtues.

In 1873 he removed to Decatur, Ga., but retained his office in Atlanta, where he daily discharged his professional duties, and also conducted the editorial and business management of the SOUTHERN MEDICAL RECORD.

On his removal to Atlanta in 1866 I met Dr. Word for the first time, and our acquaintance soon grew into a mutual and intimate friendship that bound us together until his death. My friend was not only a good man, but he had many elements of true greatness in his character, and was one of the noblest of men.

He never stooped to any corrupt policy. His principles were always pronounced for the right; hence (like all true, worthy and reliable men), could not take neutral ground upon a vital question, whether in the profession, in the church, or in public measures. He was ready at all times to vindicate truth and show up error, and never tried to make wrong triumph over right, though the unrighteous victory might promise large rewards. Steadfast and true he stood by correct principles and upheld the honor of his manhood and the profession he loved so well.

When I was requested by some of the noble, progressive men of Georgia and other Southern States to establish a new medical college in Atlanta and call it the Southern Medical College, Dr. Word, from the beginning, was the staunch and warm supporter of the enterprise. No matter what obstacle presented itself, he stood unflinchingly by his word, and gave valuable assistance to the establishment of the institution of whose faculty he was a greatly prized and honored member until the close of his life. At one time he was Dean of the Faculty, and Vice-President and Secretary of the Board of Trustees from its organization until his death. He was also Professor of Physiology in the institution from its beginning, and after the addition of the Dental Department, he was made professor of the same science in that branch of the college. His kindly, generous nature won the affection of his students, and they revered his manly integrity of character.

Dr. Word was a professional thinker, a learned and skillful physician, with conservative but progressive views in medicine, ever ready to accept new truths that would add to professional knowledge and give it an impetus towards higher attainments built upon the solid foundation of medical philosophy demonstrated by medical science. His lectures were practical and highly instructive, and often he was sublimely eloquent when speaking of life and death, the beauty and importance of medicine, and what its character should be as a science and a profession. As a writer, he was original, clear, logical and forcible, and with a mind well trained to the work. During his long association with myself as associate editor of the SOUTHERN MEDICAL RECORD, he brought that periodical up to the front rank of medical literature.

Dr. Word was (better than all else I can say of him) a Christian gentleman—a tender, sympathetic friend of humanity. During his long residence in Decatur, he endeared himself to the people of all creeds and classes, and with them as with others who knew and loved him in Atlanta, his name will be cherished with affectionate remembrance. For many years he was an elder in the Presbyterian church; also an earnest worker in the Sunday-school cause—a work that was most congenial to the pure spirituality of his Christian character. His loss is inestimable in all the positions he so faithfully filled.

A friend of God and humanity, of the rich man in his mansion and the poor man by his humble fireside, what better eulogy could be pronounced upon my dear friend and brother; what more could be said to complete the character of his well-rounded life? How I miss him in my daily walk and abors cannot be expressed by mere words. He was indeed to me a friend and brother—a friend for weal or woe, a brother always, in ready help and sympathy.

For his loss I have but one consolation—please God I shall see him again; I shall see him freed from the shackles of his feeble, pain-racked body, now cast aside like a worn-out garment, and he standing amid the glories of Paradise, clothed with the incorruptible in all its immortal strength and beauty. We will then talk together again in loving companionship, but not of how to relieve the pains and sorrows of suffering humanity, for in the City of God "there is the tree of life and its leaves are for the healing of the nations."

This, my consolation, is the only balm I can offer to the wounded hearts of his wife and children—the only hope to cheer the desolated fireside to which was wedded the affections of the husband and father when in earth. I was glad to know in my friend's lifetime that he had a beloved Christian wife, and sons and daughters who were true, noble men and women. I rejoice the more now as the knowledge gives the promise of an unbroken band in the eternal union of mother and children with him who has only gone before. I can fancy I hear him say to them and to me, as the blessed Savior said to his sorrowing disciples when about to leave them, "Yet a little while and ye shall see me."

THOS. S. POWELL, M. D.

## Figures and Tables

**Figure f1:**